# Transforming Ontology Web Language Elements into Common Terminology Service 2 Terminology Resources

**DOI:** 10.3390/jpm14070676

**Published:** 2024-06-24

**Authors:** Sara Mora, Roberta Gazzarata, Bernd Blobel, Ylenia Murgia, Mauro Giacomini

**Affiliations:** 1 UO Information and Communication Technologies, Istituto di Ricovero e Cura a Carattere Scientifico Ospedale Policlinico San Martino, 16132 Genoa, Italy; sara.mora@hsanmartino.it; 2 Healthropy Società a Responsabilità Limitata (S.R.L.), 17100 Savona, Italy; roberta.gazzarata@healthropy.it; 3 Medical Faculty, University of Regensburg, 93053 Regensburg, Germany; bernd.blobel@klinik.uni-regensburg.de; 4 Department of Informatics, Bioengineering, Robotics and System Engineering (DIBRIS), University of Genoa, 16145 Genova, Italy; ylenia.murgia@edu.unige.it

**Keywords:** ontology, CTS2, semantic interoperability, terminology resources, biomedical field

## Abstract

Communication and cooperation are fundamental for the correct deployment of P5 medicine, and this can be achieved only by correct comprehension of semantics so that it can aspire to medical knowledge sharing. There is a hierarchy in the operations that need to be performed to achieve this goal that brings to the forefront the complete understanding of the real-world business system by domain experts using Domain Ontologies, and only in the last instance acknowledges the specific transformation at the pure information and communication technology level. A specific feature that should be maintained during such types of transformations is versioning that aims to record the evolution of meanings in time as well as the management of their historical evolution. The main tool used to represent ontology in computing environments is the Ontology Web Language (OWL), but it was not created for managing the evolution of meanings in time. Therefore, we tried, in this paper, to find a way to use the specific features of Common Terminology Service—Release 2 (CTS2) to perform consistent and validated transformations of ontologies written in OWL. The specific use case managed in the paper is the Alzheimer’s Disease Ontology (ADO). We were able to consider all of the elements of ADO and map them with CTS2 terminological resources, except for a subset of elements such as the equivalent class derived from restrictions on other classes.

## 1. Introduction

The paper at hand presents an extended version of the invited paper provided to the pHealth 2022 conference [1].

**Figure 1:**
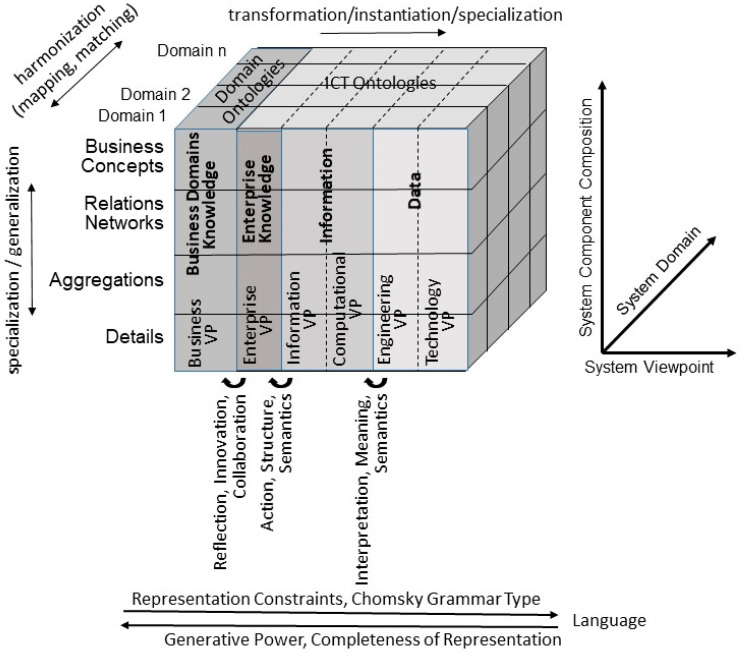
Model and framework for representing multi-domain, knowledge-based, ontology-based, and policy-driven ecosystems.

Healthcare systems are currently undergoing a transformation towards integrated, interoperable, knowledge-based, policy-driven, highly dynamic, and fully distributed ecosystems according to the personalized, preventive, predictive, participative precision (P5) medicine paradigm [2]. This requires communication and cooperation of actors from multiple disciplines with specific perspectives, contexts, objectives, using their special methodologies, languages, knowledge, and skills. The challenge of P5 medicine ecosystems is the proper representation, mapping, and matching of their domain-specific knowledge. This knowledge representation, mapping, and matching must be performed at any representation level from the real-world business system through the related IT system viewpoints, the information to represent them, and, finally, the data used, deploying more and more constrained languages from natural languages up to computational ones. Thereby, mapping between the system’s components from the perspective of different domains or different viewpoints can only be performed at a horizontal level, i.e., at the same level of granularity. To get there, components must be specialized or generalized, respectively. The corresponding representation of system-theoretical, architecture-centric, ontology-based, policy-driven multi-domain P5 ecosystems, standardized in ISO 23903 [3], is shown in Figure 1. Thereby, the Information and Communication Technology (ICT) system development process according to the ISO/IEC 10746 Reference Model Open Distributed Processing [4], just representing the five viewpoints, Enterprise Viewpoint (VP), Information VP, Computational VP, Engineering VP, and Technology VP, must be extended by the Business VP. The Business VP is an inevitable starting point for representing the real-world system and defining the requirements and objectives of the ICT system to be developed from the perspective of the involved domains experts.

While, from an ICT system development-process perspective, the transformation into the viewpoint-specific representation style is clearly defined in the aforementioned standard ISO/IEC 10746 [4]. The correct and consistent concept transformation in knowledge-driven transformed health systems is a challenge to be addressed in this paper. Thereby, we have to solve the mapping between domains represented by domain ontologies and the representational transformation of the corresponding concepts between different viewpoints. Examples for the data view representation are database management system schemas or coding systems regarding the semantical representation, terminologies, thesauri, taxonomies, glossaries, data dictionaries, or vocabularies used. The aforementioned semantical resources are collections of terms (entities) that are linked to a specific domain. They aim at creating a complete documentation that supports the correct usage of such terms.

While the domain ontologies representing the concepts of the Business VP usually deploy natural languages, the ICT VPs must be represented by more expressive, computational logic-based languages to be exploited in computing environments. The W3C Web Ontology Language OWL [5] is such a computational logic-based language that can be used to express knowledge and verify the consistency of that knowledge or to make implicit knowledge explicit [6].

Although there are many examples of transforming ontology resources (such as OWL) into other types of languages [7,8,9], the authors are not aware of any tools for organizing OWL resources using the methodology suggested by Common Terminology Service Release 2 (CTS2). This choice was made by the authors in order, on the one hand, to be able to use all of the features of ontologies encoded rigorously in OWL and, on the other hand, to be able to manage their temporal evolution through the standardized tools provided by CTS2. The need to follow the temporal evolution of said ontologies is obvious when considering ontologies defined in medical fields for which updating is rather frequent [10,11,12].

The Common Terminology Standard Version 2 (CTS2) is a HL7/OMG specification providing a generic class model and necessary interfaces for managing and sharing terminologies and ontologies by using web services. It is based on a conceptual model representing data by class models and a functional model specifying the terminology server services. The standard is distributed through the HL7 Service Functional Model (SFM) and the Object Management Group (OMG) Service Technical Model (STM), which provide both service interface specification at a functional level and technical requirements of the service. In the present paper, we use the OMG model [13].

Finally, we aimed at developing a web-based user interface that allows for the visualization and management of the terminological content of an ontology. This has been achieved using, in a standard way, functions defined using CTS2.

## 2. Materials and Methods

The purpose of this paper is to present in detail all of the choices we made to transform an ontology into CTS2 terminology resources. Therefore, a deep analysis of the key aspects, similarities, and differences of the two representations was performed.

### 2.1. Ontology Elements and Relations

An ontology is a formal explicit specification of a shared conceptualization of a domain of interest [14,15]. One widely used tool to represent ontologies within the computer science environment is the Web Ontology Language (OWL) defined by W3C [16] that is an extension of Resource Description Framework (RDF) [17] to support the definition of the semantic web. RDF is formed by two parts, as follows: the RDF model and syntax, which represents the model structure and describes the syntax, and the RDF Schema (RDFS), which describes the syntax to define the schema and vocabularies for the metadata. RDF and RDFS are used by the Simple Knowledge Organization System (SKOS) language family, created to represent glossaries, classifications, or structured vocabularies for publication purposes.

Some examples of relevant medical ontologies representing different domains are as follows: Human Phenotype Ontology (HPO) [18], Infectious Diseases Ontology (IDO) [19], and Epilepsy and Seizures Ontology (EPSO) [20]. For mapping different domain ontologies, the ISO/IEC 21838 Top-level ontologies (TLO) standard should be used [21]. In cases where a domain ontology is not available, the domain can be preliminary presented using that TLO. Within an ontology, the various objects are defined and interact with each other according to logical properties, reciprocal restrictions between and on objects, and groups and semantic sets. An ontology is characterized by three main components, as listed below.

- **Class:** Every single object belonging to the domain.
- **Annotation Properties:** Further information purely attributable to the Class itself, independent of the others. Examples of annotation properties are synonym, comment, and label.
- **Object Properties:** A restriction, which places a limit on the values of a certain Class that respects a certain Property. In most cases, the considered Property is defined within the ontology, and so its logical value scope only within it.

The interactions between the classes are defined by specific constructs called class axioms. For example, the rdfs: subClassOf construct, defined within the RDF schema and inherited by OWL, allows us to define the hierarchical relations as follows: if a Class C1 is defined as a subClassOf of another Class C2, then the set of elements that make up C1 they must at least be a subset of those that make up C2. A Class is therefore, by definition, a subClass of itself, as the subset can also be the whole set.

Another class axioms is the owl:equivalentClass construct, which indicates that the set of elements of Class C1 are equivalent to those of Class C2. Therefore, the two sets must have exactly the same number and the same elements.

### 2.2. CTS2 Terminology Resources and Profiles

**Figure 2:**
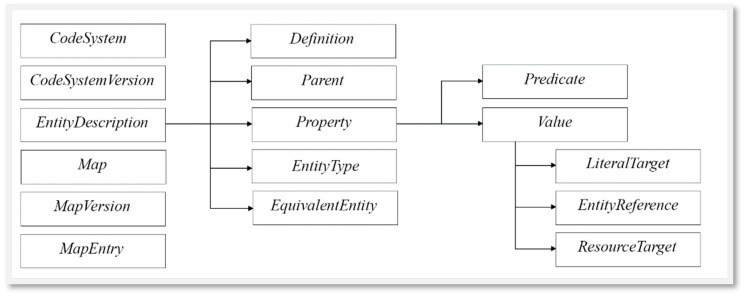
The CTS2 terminology resources and the detail of the EntityDescription elements considered in this paper.

The main CTS2 terminology resources involved in this process are as follows: CodeSystem, CodeSystemVersion, EntityDescription, Map, MapVersion, and MapEntry (Figure 2).

**A CodeSystem** can be a classification system, a code system, an ontology, or a thesaurus, etc. Together with some identifying information such as the name, it includes information about the publisher, release cycles, purpose, etc. However, as this kind of terminological resources may evolve over time, it is necessary to manage the versioning option. Therefore, to each CodeSystem may correspond one or more **CodeSystemVersions** containing information about release date, release format, contact information, etc. Then, each CodeSystemVersion contains one or more **EntityDescriptions** describing a class, a role, or an individual from the specific CodeSystemVersion.

The EntityDescription is the most complex but also the most interesting item to investigate. During the translation process, to maintain the characteristics of the specific element of the ontology, it was necessary to include different metadata. Among others, we considered the following (Figure 2):

- **Definition:** An explanation of the intended meaning of a concept. An EntityDescription may have multiple definitions, each derived from a different source, represented in a different language or having a different purpose.
- **Parent:** The set of direct “parents” defined in the same CodeSystemVersion. It is the responsibility of the service to determine what predicate(s) represent “parent/child” relationships.
- **Property:** Additional “non-semantic” (annotation) assertions about the entity being described that do not fit into the other categories.
- **EntityType:** The set of type(s) a resource can take, and it should include owl:Class, owl:Individual, rdf:Property, or skos:Concept, although it may carry many other types as well.
- **EquivalentEntity:** An entity that has been determined to be equivalent to the about entity in the context.

The element we mainly focused on is **Property** and its two main components are as follows:

- **Predicate:** The name or URI of the property predicate. It can be literal or an EntityDescription itself, namely, an Annotation Property or an Object Property.
- **Value:** The target(s) of the property. Note that this can only represent the literal format of the property. The details about the original property will be found in the *CorrespondingStatement* if the CTS2 implementation supports the statement profile. So, the attribute value of a property is of Class *StatementTarget*, and it can be from three of the following types:
  ○ Literal Target: When the statement type is LITERAL. It can be used for properties like the entity “label” or “comment”.
  ○ Entity Reference Target: The URI and optional namespace/name when the target type is ENTITY. It can be used when a property refers to another entity.
  ○ Resource Target: When the statement type is RESOURCE.

An entity may have more than one value for the same predicate, so it is necessary to create a list of *StatementTargets* containing all the items and then assign the list to *Property.Value*, while *Property.Predicate* remains unchanged.

Finally, it could be necessary to link resources across CodeSystems and CodeSystemVersions. To this aim, the CTS2 standard provides the following three terminology resources: Map, MapVersion, and MapEntry. A **Map** is a collection of rules necessary to transform entities of a CodeSystem into others represented in a second one. It also includes information about creators, intended use, CodeSystem involved, etc. As mentioned previously, it is necessary to deal with changes over time in the terminological resource. Therefore, it is possible to identify a specific version of the Map, called **MapVersion**. Then, to each MapVersion, correspond one or more MapEntries, i.e., the definition of a set of rules identifying how a single Entity that belongs to the original CodeSystemVersion maps onto null, one, or more target Entities that belong to the destination CodeSystemVersion.

The CTS2 standard defines, for each terminology, resources (also known as structural profiles) and different functional profiles. The most important ones for this paper are the following: Read, Query, Update, and Maintenance (which has the capability to create resources). The CTS2 specification defines the implementation of a specific server for each couple of structural profile/functional profile (for example, CodeSystem Catalog Maintenance).

### 2.3. Translation Process Pipeline

The ontology that we considered testing our mapping system on was the Alzheimer’s Disease Ontology (ADO) [22], a knowledge-based ontology which encompasses concepts related to Alzheimer’S Disease. In order to show how an ontology can be mapped with another terminology using CTS2, the Analitica Avanzata su Dati Complessi (ADA Lab), a more general ontology, was used to model relations in a complex and interdisciplinary environment [23].

The pipeline of the translation process is composed by the following main steps.

**Create CodeSystem (I)** using the CodeSystem Catalog Maintenance Service functional profile. Within this phase, we provided the following input parameters: *Uniform Resource Identifier* (URI), i.e., an external link of the resource; and *Name*, i.e., the identifier of the catalog that we want to create, to use locally (Enterprise VP).
**Create CodeSystemVersion (II)** using the CodeSystemVersion Catalog Maintenance Service. Equally to the CodeSystem, one of the input parameters is *Name*, which uniquely identifies the specific version in the CodeSystem. The other input parameter that we considered is *VersionOf*, which contains the name or URI of the CodeSystem that the version belongs to (Information VP).
**Create EntityDescription (III)** using the Entity Description Maintenance Service. For each entity, we set two important input parameters, as follows: *EntityID*, i.e., the entity code and/or namespace identifier; and DescribingCodeSystemVersion, which contains the URI or local identifier of the CodeSystemVersion this entity belongs to (Computational VP).
**Update EntityDescription (IV)** using the Entity Description Maintenance Service. We completed each entity with all of the information linked to the single ontology class or property. In general, the components of an ontology resource can be distinguished in the following: (i) the components directly mappable into an element of the CTS2 EntityDescription, as displayed in Table 1; (ii) the components that could not fit into any predefined item, but which can be mapped into properties, as displayed in Table 2; and (iii) the components that could not fit into any predefined item, but which can be mapped into a CTS2 terminology resource, i.e., the properties devoted to Map elements among ontologies and/or other coding systems (Engineering VP).

**Table 1:** Correspondence between elements of an ontology class that can be directly mapped to the CTS2 EntityDescription components.

Ontology Concept Metadata	CTS2 Resource Metadata	Data Type
Example	.Example	List (0…N)
IsDefinedBy	.Definition	List (0…N)
SubClassOf	.Parent	Class (1…N)
EquivalentClass	.EquivalentEntity	Class (0…N)

**Table 2:** Four cases to which all of the elements of an ontology class/property not contained in Table 1 can belong to, as follows: (a) the predicate is a text and the value is a text, (b) the predicate is a text and the value is an entity, (c) the predicate is an entity and the value is a text, and (d) the predicate is an entity and the value is an entity.

	Value Is a Text	Value Is an Entity
**Predicate is a text**	The **predicate** contains the name and namespace of the statement predicate (*type: EntityNameOrURI*). The **value** element is a statement target of type LITERAL (*type: Opaque-Data*).	The **predicate** contains the name and namespace of the statement predicate (*type: EntityName-OrURI)*. The **value** element is a statement target of type ENTITY *(type: EntityNameOrURI*).
**Predicate is an Entity**	The **predicate** contains the URI of the entity of type Annotation Property (*type: EntityNameOrURI*). The **value** element is a statement target of type LITERAL (*type: Opaque-Data*).	The **predicate** contains the URI of the entity of type Object Property (*type: EntityNameOrURI*). The **value** element is a statement target of type ENTITY (*type: Entity-NameOrURI*).

**Create MapCatalogEntry (V)** using the Map Catalog Maintenance Service. We provided the following input parameters: *MapName*, i.e., the name the new entry will be known as within a local context; *FromCodeSystem*, i.e., the name or URI of the CodeSystem that the “from” entities belong to; and *ToCodeSystem*, i.e., the name or URI of the CodeSystem that the “to” entities belong to.
**Create MapVersion (VI)** using the Map Version Maintenance Service. The considered input parameters are as follows: *MapVersionURI*, i.e., the state of the resource version which can be “OPEN” or “FINAL”, and once the MapVersion is finalized it becomes immutable; *MapVersioneName*, i.e., an identifier to uniquely identify the MapVersion in a local context; *FromCodeSystemVersion*, i.e., the identifier (name or URI) of the specific CodeSystemVersion that the “from” entities belong to; and *ToCodeSystemVersion*, i.e., the identifier (name or URI) of the specific CodeSystemVersion that the “to” entities belong to.
**Create MapEntry (VII)** using the Map Entry Maintenance Service. This is a set of mappings having the same entity identifier as *MapFrom*.
**Update MapEntry (VIII)** using the Map Entry Maintenance Service. After we defined the entity of the “FROM” side, it is necessary to define one or more entities on the “TO” side, e.g., ones belonging to other CodeSystems. To perform that, the following two operations need to be executed.
  **Add MapSet (VIII.i)**. Specifically, each MapEntry may contain one or more MapSets, defining rules and characteristics of the Map. Considering each Mapset, it is necessary to perform the function below.
  **Add MapTarget (VIII.ii)**. In detail, the item MapTarget identifies the entity to include it in the Map on the “TO” side.

## 3. Results

The architecture that we used to develop the transformation of a semantical resource from an OWL standard format to a CTS2 standard resource allows the user to visualize and manage the terminological content of an ontology, and is formed by the following three components: a CTS2-compliant service, a ASP.NET Console Application (.NET Framework), and a ASP.NET Web Application (.NET Framework).

**Figure 3:**
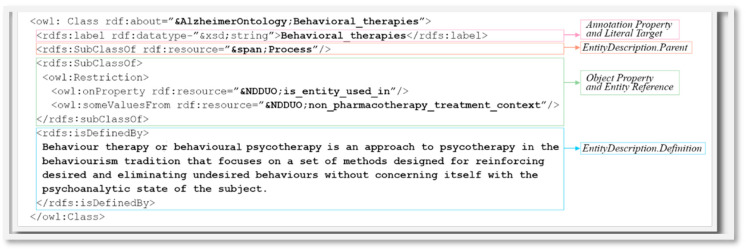
An example of an OWL class defined in ADO, with an indication of the corresponding CTS2 EntityDescription element in which every OWL element can be mapped.

The CTS2-compliant service is represented by HQuantum© Technology Service (HTS) powered by Healthropy s.r.l. [24,25]. In this way, HTS provides a standard interface to access to read, query, and manage terminological content into the database where the ontology has been stored. The ASP.NET Console Application (.NET Framework) is a client of HTS and was implemented to allow for the first import of an OWL ontology into the HTS, as was made for the Alzheimer’s Disease Ontology (ADO). In fact, this application was used to create the CodeSystem “ADO” (by calling CTS2 operation Code System Catalog Maintenance Service/CreateCodeSystem) and its first version “ADPV1” (by calling CTS2 operation Code System Version Catalog Maintenance Service/CreateCodeSystemVersion) and each EntityDescription. In detail, the application opens the XML, which contains the ontology, and, for each OWL class, converts it to the corresponding CTS2 EntityDescription, creates it in HTS (by calling CTS2 operation Entity Description Catalog Maintenance Service/CreateEntityDescription), and updates it by indicating all of the needed EntityDescription elements, as described in Section 2 (by calling CTS2 operation Entity Description Catalog Maintenance Service/UpdateEntityDescription). In Figure 3 is represented an example of the OWL class “Behavioral_therapies”, defined in the ADO. Following the same process, also, the “AdaLab” CodeSystam, its first version “AdaLab1”, and some EntityDescriptions were uploaded in HTS.

The Console Application was adopted to store, in HTS, some examples of maps between the entities of “ADOV1” and “AdaLab1” available in an Excel spreadsheet which contains two rows: one for the EndityDesctiption source (i.e., the entity defined in ADOV1), and another one for the EndityDesctiption target (i.e., the entity defined in AdaLab1). In fact, this application was used to create the MapCatalogEntry “FromADOToAdaLab” (by calling CTS2 operation Map Catalog Maintenance Service/createMapCatalogEntry) and its first version “FromADOV1ToAdaLab1” (by calling CTS2 operation Map Version Maintenance Service/CreatemapVersion) and each MapEntry. In detail, the application opens the Excel spreadsheet, which contains the maps, and for each row, converts it to the corresponding MapEntry, creates it in HTS by indicating the entity source (by calling CTS2 operation Map Entry Maintenance Service/CreateMapEntry), and updates it by indicating the entity target, as indicated in Section 2 (by calling CTS2 operations Map Entry Maintenance Service/addMapSet and AddMapTarget).

Once an ontology is created in HTS, it is available to the user through the ASP.NET Web Application (.NET Framework), which, after login, can access four main sections for each CodeSystem: Read, Query, Maintenance, and Map.

**Figure 4:**
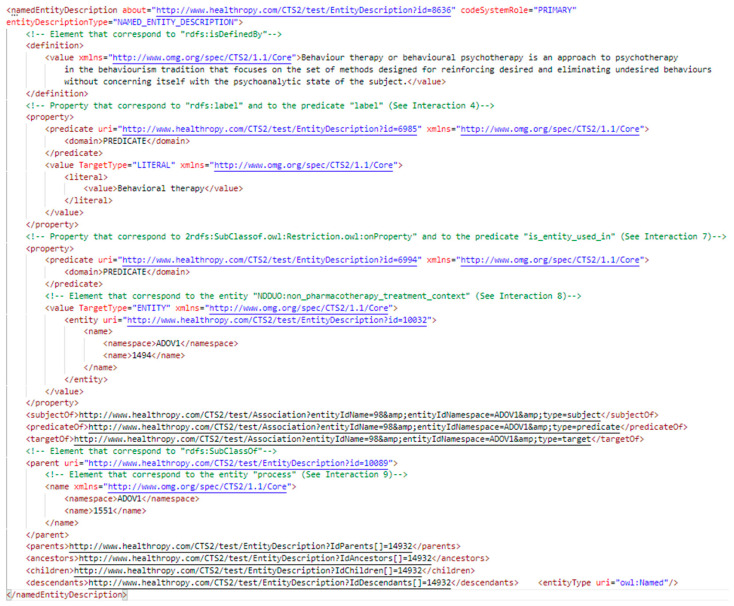
An extract from the EntityDescription “Behavioral therapies” defined in the ADOV1 contained in the SOAP message, intercepted as a response of the CTS2 operation Entity Description Read Service/read.

**Figure 5:**
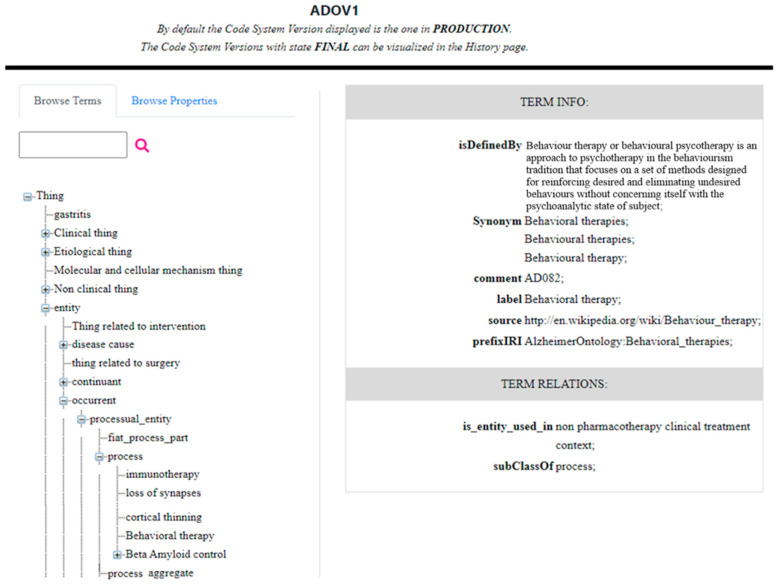
An example of the tree visualization of ADO classes (**left**) and entity details (**right**).

The Read section of the web application allows the user to browse all of the entities stored within the HTS, both classes and properties, through a tree-view visualization or a term search. The tree view presents the concepts following the hierarchical organization in the ontology through the CTS2 EntityDescription elements Parent or Children. To fill the elements of this web object, the application interacts with HTS by calling the CTS2 operations provided by the Entity Description Read Service. By clicking on an element of the tree view, it is possible to obtain all of the related details. In Figure 4, there is presented an extract of the EntityDescription, which corresponds to “Behavioral therapies” defined in the ADOV1, and is contained in the SOAP message intercepted as a response of the read operation of the Entity Description Read Service. In the details are represented the CTS2 elements that correspond to the OWL elements reported in Figure 3. Figure 5 represents the tree view and the details for the same entity, “Behavioral therapies”. It is possible to see, in “Term Info”, the OWL elements isDefinedBy and the label “Term Info”, and is_entity_used_in and subClassOf in “Term Relations”, with the same value as presented in Figure 3. As indicated in Section 2, the OWL element subClassOf “process” represents the parent of “Behavioral therapies”, as represented in the tree view. The complete CTS2 EntityDescription “Behavioral therapies”, and the other relevant EntityDescriptions, needed to fill the webpage presented in Figure 5, as is reported in the Supplementary Materials.

**Figure 6:**
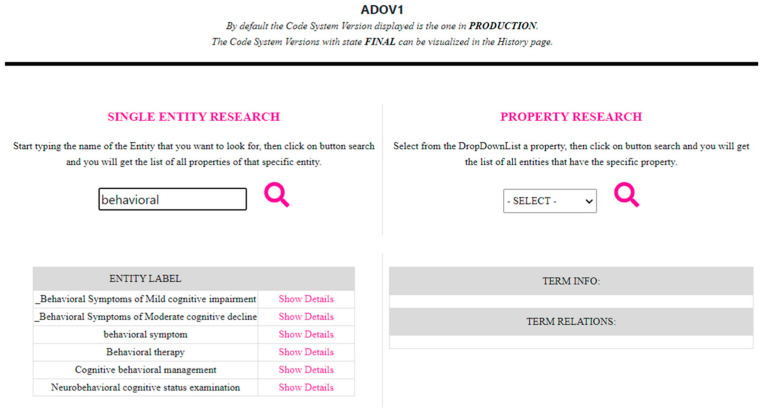
An example of a search for a name.

The Query section allows the user to search entities within a CodeSystem stored in HTS. The user can search for entities that contain a specific string in the name or for ones that have a specific property. To execute these searches, the web application calls the CTS2 operations Entity Description Query Service/Restrict and ResolveAsList. An example of a search is presented in Figure 6. All of the resulting entities are presented in the Table “Entity Label”, and the user can visualize the details of a specific entity by clicking on “Show Details”, which will be presented to the user in the same way as represented in Figure 5.

**Figure 7:**
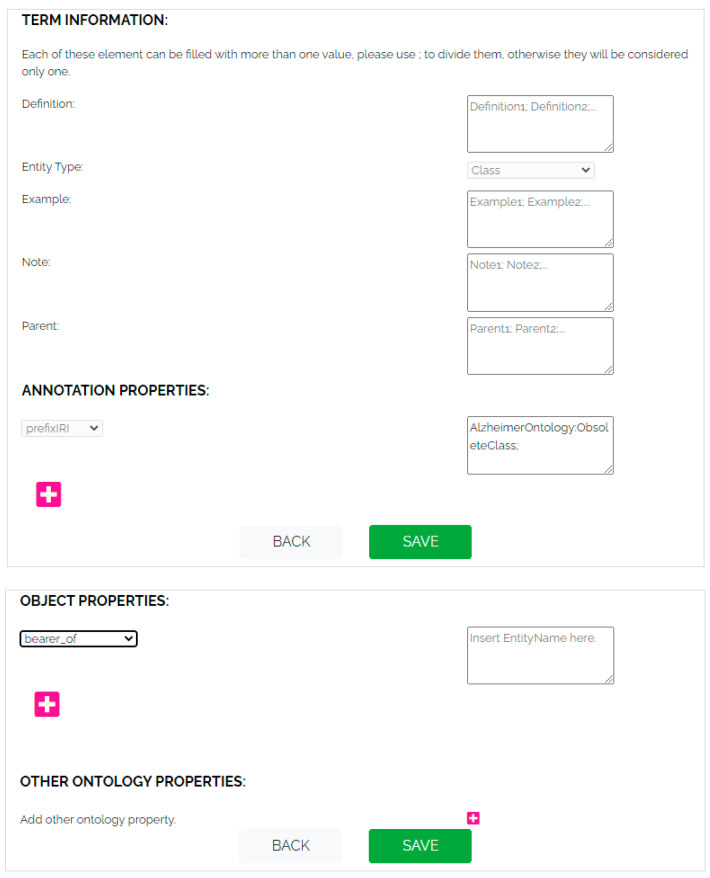
Visualization of term information, annotation properties, and object properties.

The Maintenance section allows the user to modify the current CodeSystemVersion (i.e., the version of the CodeSystem in production) in HTS. In detail, the user can update information about the CodeSystemVersion or close it (by calling CTS2 operation Code System Version Maintenance Service/updateCodeSystemVersion) and then open a new one (by calling CTS2 operation Code System Version Catalog Maintenance Service/CreateCodeSystemVersion). He/she can also manage the entity defined in the current version by creating a new entity or by updating or deleting an existing one (by calling CTS2 operation Entity Description Catalog Maintenance Service/CreateEntityDescription and/or UpdateEntityDescription). Figure 7 shows how a user can add information and properties to a new entity by adopting the web application.

**Figure 8:**
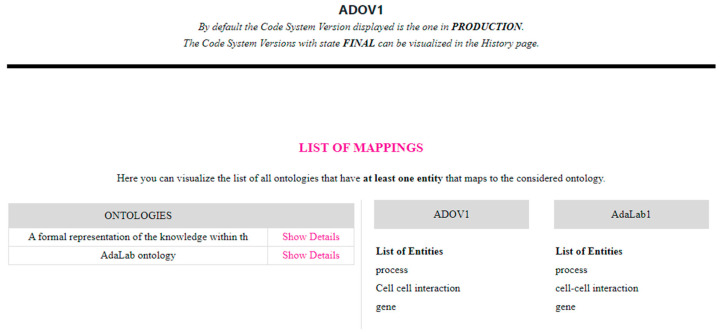
List of all MapEntries belonging to the MapCatalog identified by the two CodeSystems ADO and AdaLab, and more in detail, the two CodeSystemVersions named ADOV1 and AdaLab1.

The last functionality provided by the web application is the Map section, which allows the user to visualize all existing mappings between the current version of a specific CodeSystem and the current version of another CodeSystem stored in HTS. The client application interacts with HTS to obtain all of the Maps that are defined for the specific source CodeSystem (by calling CTS2 operations Map Catalog Query Service/restrictByCodeSystem and resolveAsList) and propose a list of the target CodeSystems, which correspond to the Maps, to the user on the left side of the webpage in the Ontologies section (Figure 8). The user can select the target CodeSystem, and by clicking on “Show Details”, the website interacts with HTS to retrieve all of the MapEntries defined for the current MapVersion (by calling CTS2 operations Map version Query Service/restrictToCodeSystems and resolveAsList) and present them to the user. Figure 8 shows an example of the three MapEntries defined in the MapCatalogue that correspond to the current version of ADO and AdaLab ontologies.

## 4. Discussion

Considering the scenario of data integration, the development and usage of standard terminologies obtained a primary role and consistently improved the quality of the resulting outcome. However, it should be considered that terminologies evolve, e.g., the list of terms constantly undergo updates, including insertions and deletions. Therefore, it is necessary to adopt a solution able to track all changes in order to ensure and maintain the integrity of the terminology resource. Following the Chomsky hierarchy of language grammars, we should not forget that an ontological representation is richer and more consistent than a representation at a lower level, such as a terminology (see Figure 1). Therefore, it is inevitable to check the completeness and consistency of mapping two different representation styles using the model and framework of ISO 23903 [3].

OWL represents the most suitable solution to distribute an ontology, but it was not created for the purpose of tracking and visualizing the changes of each concept over the time. For this purpose, the concept of a terminology service was created to indicate a tool that allows us to provide access to terminology content through interfaces to read, query, maintain, and visualize the history of a specific terminology resource. The recent possible standard solution to define a terminology service, Application Programming Interface (API), is represented by the HL7 Fast Healthcare Interoperability Resources (FHIR) Terminology Service, already adopted in the literature [26,27,28,29]. The main limitation of the present approach is the possibility of establishing only hierarchical relations between concepts defined in the same code system and those mapped between concepts defined in different code systems. In addition, the FHIR Terminology Service allows us to work on the overall code system, rather than directly on the single entity, making the history of the changes in a single concept difficult to retrieve and visualize. On the contrary, a possible non-standard solution is Protégé (https://protege.stanford.edu/ accessed on 20 June 2024). Specifically, it is an open-source tool aimed at supporting the creation and management of ontologies, and it automatically tracks the changes, made available in the revision history. Its main advantage is that, as it is specifically devised for ontologies, the system is able to deal with all components of an ontology [21,30]. It also presents a main limitation, being that, to the best of our knowledge, at present, there is no API available allowing for a rich set of operations for ontology creation and management. Therefore, users can only use the web interface (Web Protégé) to interact with the database, thus requiring an intense human effort.

In our work, we chose to address the problem of terminology management with a standard solution, specifically the Common Terminology Service Release 2 (CTS2), because it is services-based and we have already used it in several scenarios [13,24,25,26,31,32]. As a main advantage, the use of CTS2 standard provides specifications useful to implement a set of operations to completely manage all aspects of CTS2 resources. CTS2 has some feature that are suitable for our context of use. First of all, the CTS2 information model for the concept (i.e., Entity Description) provides an indication not only of the direct parents and children, but also for the ancestors and descendants. This is useful to provide an easy navigation in the ontology, as we proposed with a tree view. In addition, the CTS2 information model works at an atomic level (i.e., single concept, Entity Description, and map, MapEntry), making the history of the changes in a single concept and map easy to retrieve and visualize, an important aspect for the authoring of an ontology. For this reason, for a future work, we are planning to implement new functionalities on the web application to visualize the history of the single concept and maps, adopting the operation provided by the CTS2 Entity Description History Service and Map Entry History Service. Other future work will be made on the side of the terminology service and on the client applications. The authors intend to implement the CTS2 Entity Description Transform Service, which is not yet available on HTS, to allow us to transform a concept represented in OWL in the corresponding CTS2 Entity Description. This will allow us to make the translation at the service level instead of at the client level, guaranteeing a more efficient control on the process. Finally, the authors intend to integrate the functionalities provided by the console application to the web application in order to have one tool to perform all of the operations on an ontology.

In personalized medicine, it is essential to accurately describe the situation of a patient at all levels (from macroscopic [22] to molecular [33]) [34,35]. These kinds of systems combine, on the one hand, the analytical nature of ontologies and, on the other hand, the systematic approach and rigor used in standardized tools, such as the CTS2, to preserve the historical evolution of the terminological systems. Therefore, we believe that it can be considered to be one of the enabling tools for the real implementation of the paradigms of medical personalization.

## Data Availability

All data is publicly available at: http://www.medinfo.dibris.unige.it/VBC_CTS2/ (accessed on 20 June 2024).

## Supplementary Materials

The following supporting information can be downloaded at: https://www.mdpi.com/article/10.3390/jpm14070676/s1, File S1: XML file with the complete CTS2 EntityDescription of “Behavioral therapies”.
